# Effects of the Polar Fraction of *Lophocereus schottii* on Gene Expression and Hepatocyte Proliferation in a Wistar Rat Model of Hepatocellular Carcinoma

**DOI:** 10.3390/ijms26199788

**Published:** 2025-10-08

**Authors:** Marina Campos-Valdez, Jaime Sánchez-Meza, Arturo Orozco-Barocio, José A. Domínguez-Rosales, Juliana Marisol Godínez-Rubí, Sarai C. Rodríguez-Reyes, Erika Martínez-López, Miriam R. Bueno-Topete, Manuel A. Castro-García, Guillermo M. Zúñiga-González, Daniel Ortuño-Sahagún, Laura V. Sánchez-Orozco

**Affiliations:** 1 Instituto de Investigación en Enfermedades Crónico Degenerativas, Centro Universitario de Ciencias de la Salud, Universidad de Guadalajara, Guadalajara 44340, Jal., Mexico; campos.ibt@gmail.com (M.C.-V.); jaimesanchez96@hotmail.com (J.S.-M.);; 2 Laboratorio de Inmunobiología, Departamento de Biología Celular y Molecular, Centro Universitario de Ciencias Biológicas y Agropecuarias, Universidad de Guadalajara, Zapopan 44600, Jal., Mexico; arturo.obarocio@academicos.udg.mx; 3 Laboratorio de Patología Diagnóstica e Inmunohistoquímica, Departamento de Microbiología y Patología, Centro Universitario de Ciencias de la Salud, Universidad de Guadalajara, Guadalajara 44340, Jal., Mexico; juliana.godinez@academicos.udg.mx; 4 Instituto de Nutrigenética y Nutrigenómica Traslacional, Centro Universitario de Ciencias de la Salud, Universidad de Guadalajara, Guadalajara 44340, Jal., Mexico; 5 Laboratorio de Mutagénesis, Centro de Investigación Biomédica de Occidente, Instituto Mexicano del Seguro Social, Guadalajara 44340, Jal., Mexico; 6 Laboratorio de Neuroinmunobiología Molecular, Instituto de Investigación en Ciencias Biomédicas, Centro Universitario de Ciencias de la Salud, Universidad de Guadalajara, Guadalajara 44340, Jal., Mexico; daniel.ortuno@academicos.udg.mx

**Keywords:** *Lophocereus schottii* polar fraction, hepatocellular carcinoma, hepatocytes proliferation, *TGFB1*, *AFP*, *CYP2E1*, *CAT*, *SOD*, *COL1A*

## Abstract

Hepatocellular carcinoma (HCC) remains a major global health problem for which there are few effective treatments. Phytochemicals from natural sources, such as those found in cacti, exhibit chemoprotective and hepatoprotective properties. In this study, the effect of the polar fraction of *Lophocereus schottii* (LsPF) was investigated in a Wistar rat model of HCC induced by weekly administration of diethylnitrosamine (DEN, 50 mg/kg, i.p.) and 2-acetylaminofluorene (2-AAF, 25 mg/kg, i.g.) for 13 weeks. LsPF (50 mg/kg, i.g., three times per week) was administered either concurrently with HCC induction beginning in the first week or after seven weeks of HCC induction. LsPF did not lead to a significant improvement in macroscopic, biochemical or histologic results. However, when LsPF was administered after 7 weeks of HCC induction, it modulated the expression of genes related to liver carcinogenesis, including *SOD*, *CAT*, *CYP2E1*, *TGFB1*, *AFP*, and *COL1A.* In addition, co-administration of LsPF along with the damage treatment decreased the number of mitotic hepatocytes. These results suggest that LsPF can modulate gene expression and hepatocyte proliferation in HCC, with efficacy depending on the timing of administration, disease stage, and administration method. Further studies are needed to optimize its therapeutic potential.

## 1. Introduction

According to the Global Cancer Observatory, in 2022, liver cancer occupied the sixth place in incidence (866,136 new cases) and the third place in mortality from cancer (758,725 deaths) [1]. The number of liver cancer cases is expected to increase by 55% (comparing 2020 to 2040); this projected increase could drive the demand for resources to care for liver cancer patients [2]. Of all primary liver cancer cases, hepatocellular carcinoma (HCC) accounts for 80%, intrahepatic cholangiocarcinoma for 14.9%, and other specified histology for 5.1% [3].

The etiology of liver cancer is influenced by various factors that differ from region to region. In Asia and Africa, aflatoxin exposure and hepatitis B virus infection remain important factors, while in Western countries, new risks such as metabolic dysfunction-associated fatty liver disease are more prevalent [4]. However, the trend shows a transition from viral to non-viral causes and an increasing incidence of HCC associated with alcoholic and non-alcoholic fatty liver disease, with non-alcoholic steatohepatitis being the fastest-growing etiology [5,6].

Animal models of liver cancer in rats (*Rattus norvegicus*) are useful for the investigation of new compounds that may be potential anticancer alternatives. The advantage of chemically induced models is their similarity to the cycle of injury, fibrosis, and malignancy observed in humans [7].

N-nitrosodiethylamine (DEN) is a genotoxic agent that is carcinogenic by alkylating DNA and generating reactive oxygen species (ROS) via the activation of cytochrome P450 (CYPs) in hepatocytes [8]. Oral administration of N-2-fluorenylacetamide (2-AAF) leads to tumors in various tissues of mice and rats, including the liver (HCC or cholangiocarcinoma) [9]. In addition, 2-AAF promotes DEN-induced precancerous lesions of the liver by decreasing autophagy, apoptosis, the expression of tumor suppressor genes and increasing cell proliferation [10]. Chronic administration of DEN and 2-AAF leads to HCC, which is characterized by fibrogenesis, inflammation, antioxidant, and hepatic system failure [11].

An increase in pro-inflammatory cytokines such as interleukin (IL)-6, IL-1β, IL-17, and tumor necrosis factor α (TNF-α) leads to cell damage and plays a key role in the development and progression of primary liver cancer [12,13]. Chronic inflammation is associated with an increase in ROS, which can induce mutations in nuclear and mitochondrial DNA, and promote genomic instability and epigenetic changes [14]. Subsequent hepatic regeneration increases the probability of errors in DNA replication. If liver damage persists, elevated levels of pro-inflammatory cytokines and ROS perpetuate the liver damage, characterized by persistent inflammation, oxidative damage, fibrosis, DNA damage, and ultimately, the development of HCC [13].

Hepatic stellate cells (HSC) play a pivotal role in the development of fibrosis. These cells regulate the secretion of extracellular matrix (ECM) proteins, matrix metalloproteinases, and their specific tissue inhibitors. Following persistent liver injury, HSC are activated and undergo transdifferentiation into myofibroblasts. This transformation represents a turning point in fibrogenesis, as the activated cells exhibit a profibrotic phenotype characterized by sustained production of collagen and fibronectin. Furthermore, they secrete transforming growth factor beta (TGF-β), a central cytokine that triggers the production of collagen 1 (COL1). Beyond its role in fibrosis, TGF-β also contributes to the proliferation, migration, and regeneration of hepatocytes through the modulation of diverse signaling pathways, highlighting its dual role in both repair processes and injury [15].

In addition to genetic alterations caused by oxidative damage to nuclear and mitochondrial DNA, ROS also disrupt the tumor microenvironment, which in turn regulates the progression of HCC. The cellular redox balance is maintained by a system of antioxidant enzymes. This system includes superoxide dismutase (SOD), which catalyzes the dismutation of superoxide anions (O_2_^−^) into hydrogen peroxide (H_2_O_2_), which is converted to H_2_O by catalase (CAT) and glutathione peroxidase (GPX) [14]. Furthermore, inducible nitric oxide synthase (INOS) is inactive under physiological conditions but is upregulated in response to inflammatory stimuli, leading to the production of nitric oxide (NO). The reaction of NO with O_2_ or O_2_^−^ is associated with cancer pathogenesis, as it promotes nitrosative and oxidative stress through nitration and oxidation reactions that damage cellular components [16].

Novel therapeutic approaches may attempt to target these interconnected cellular processes by inhibiting ROS-mediated effects, such as the activation of pro-inflammatory and profibrogenic signaling pathways, as well as the modulation of key antioxidant responses, to counteract oxidative stress-induced damage [17].

Most of the currently approved systemic treatments for HCC are tyrosine kinase inhibitors, vascular endothelial growth factor inhibitors, and immunotherapeutic agents. The first-line agents are sorafenib, lenvatinib, atezolizumab plus bevacizumab, durvalumab plus tremelimumab, and durvalumab alone. The second-line agents are regorafenib, ramucirumab, and cabozantinib. The synergy of sequential/combined systemic therapies and locoregional therapies offers clinical benefits, including prolongation of overall survival in early, intermediate, and advanced HCC. For these reasons, combining treatments with different mechanisms of action is often the easiest way to overcome problems with drug resistance [18,19,20]. The increasing incidence of HCC and the still high mortality rate draw attention to the search for new therapeutic solutions.

Bioactive substances (such as polysaccharides, alkaloids, phenols, peptides, and active bacteria/fungi) have a significant anti-tumor effect and have fewer side effects than other chemotherapy drugs. They can also enhance the cytotoxicity of chemotherapy drugs in cancer cells and protect healthy cells from the adverse reactions caused by chemotherapy drugs. The effects of these substances against liver cancer include regulation of signaling pathways related to apoptosis and autophagy, inhibition of cancer cell growth, proliferation, and/or migration, regulation of the cell cycle of cancer cells, and regulation of the immune response [21].

Cacti typically contain considerable amounts of dietary fiber, vitamins (including vitamins A, C, and the B-complex), minerals (such as calcium, magnesium, and potassium), natural pigments, terpenes, alkaloids, phenolic compounds, and antioxidants; additionally, various extracts from several cacti have anticancer potential [22]. Those compounds derived from the cactus family modulate pro-inflammatory cytokines and signaling pathways and could thus mitigate liver inflammation associated with various liver diseases. In addition, preclinical studies suggest that cactus extracts or their isolated compounds exhibit hepatoprotective effects against toxic effects (including chemically induced hepatotoxicity). These compounds could promote liver regeneration, inhibit fibrosis, and support detoxification mechanisms [23].

*Lophocereus schottii* (Engelm.) Britton & Rose Cactaceae (also known as “senita cactus”) grows 2 to 4 m tall, has 5 to 10 ribs, and white to pale pink flowers. *L. schottii* consists of three taxonomic varieties: *L. schottii* var. *australis*, *L. schottii* var. *schottii,* and *L. schottii* var. *tenuis;* they grow in the northwest of Mexico (Sonora and the peninsular desert, in large parts of Baja California, and the adjacent continental desert). The stem of this cactus is traditionally used to heal wounds, sores, stomach ulcers, stomach cancer, and diabetes [24,25].

Orozco-Barocio et al. have reported that the ethanolic extract reduces tumor volume in a solid L5178Y mouse lymphoma model and that the ethanolic extract and its polar fraction have a cytotoxic effect on cells undergoing mitosis (cell line L5178Y murine lymphoma cells and splenocytes stimulated with concanavalin-A). The effect could be due to alkaloids, steroids, flavonoids, terpenoids, phenols, and quinones (in the case of the polar fraction, steroids and quinones are not present). We have previously characterized the putative secondary metabolites in the polar fraction of *L. schottii* (LsPF) by UPLC [26,27] being mainly: lophocerine [28], peyophorine [29], phenylalanine-betaxanthin [30] (phenylalanine-bx), 2,17-didecarboxy-neobetanin, kaempferol xiloside, kaempferol 3-O-glucoside (Astragalin) [31], cyanidin 3-O-glucoside (Kuromanine) [32], kaempferol 3-O-xylosyl-glucoside [33], phytofluene, alpha-tocotrienol [34], coumaroyl malic acid [35] and a small portion of unidentified compounds.

The aim of this study was to evaluate the effect on the proliferation of Huh-7 and HepG2 cell lines and to determine the efficacy of long-term (13 weeks) versus short-term (7 weeks) LsPF treatment in a chemically induced model of HCC in Wistar rats using DEN and 2-AAF.

## 2. Results

### 2.1. Identification of Compounds from the Polar Fraction Extract

The characterization of the secondary metabolites identified by UPLC-MS in the LsPF is shown in Supplementary Table S1 and described in Orozco-Barocio et al. (2022) [27]. The results of the qualitative analysis of LsPF from different extractions are described in Supplementary Table S2.

### 2.2. LsPF Reduces the Viability of Hepatocellular Carcinoma Cells In Vitro

**Figure 1:**
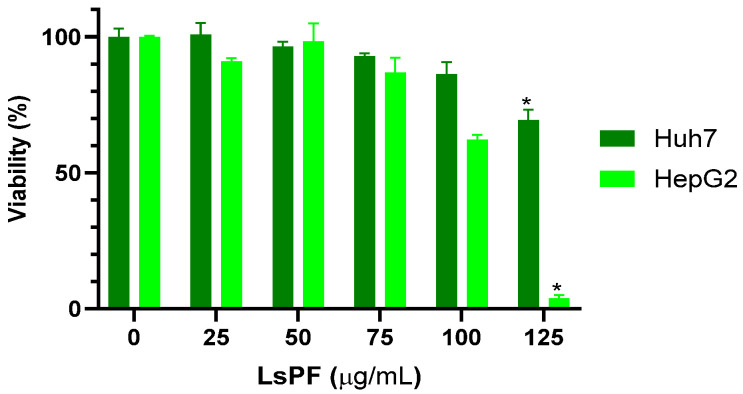
LsPF reduces the viability of hepatocellular carcinoma cells in vitro. Data expressed in viability percentage (± SEM). * *p* < 0.05 compared to the 0 µg/mL group using the Kruskal–Wallis test with Dunn’s multiple comparison test; statistical significance was calculated using corrected ABS values. [µg, micrograms; mL, milliliters].

After 72 h of exposure to LsPF, the viability of Huh7 and HepG2 cells started to decrease at a dose of 75 µg/mL, with the effect being more pronounced in HepG2 cells. However, the decrease in viability was significant at a dose of 125 µg/mL of LsPF, reducing the viability of Huh-7 cells to 69.5% and HepG2 cells to 3.8% compared to cells without treatment (*p* < 0.05) (Figure 1), indicating a reduction in the proliferation of HCC cells due to exposure to high doses of the fraction.

### 2.3. Chemical Damage Reduces the Weight Gain of Animals Without Compromising Survival

**Figure 2:**
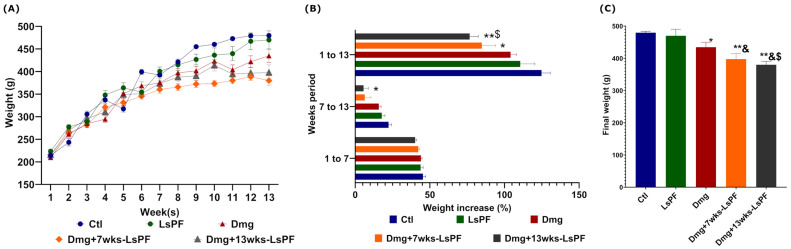
Chronic administration of DEN and 2-AAF with or without LsPF tends to result in less weight gain. (**A**) Weekly mean weight (± SEM). (**B**) Mean (± SEM) percentage weight gain [Calculated as: [(Final weight-Initial weight)/Initial weight) × 100] at 1 to 7, 7 to 13, and 1 to 13 weeks, statistical significance was analyzed by 2-way ANOVA and contrasts by Tukey’s multiple comparison test; (**C**) Final mean weight (±SEM) of the groups, statistical significance was analyzed by Mann–Whitney test. * *p* < 0.05; ** *p* < 0.01 compared to the Ctl group; ^&^ *p* < 0.05 compared to the LsPF group. ^$^ *p* < 0.05 compared to the Dmg group. [Ctl, Control; LsPF, *Lophocereus schottii* Polar Fraction; Dmg, Damage; wks, weeks; g, grams].

The weight of the animals was monitored during the entire 13-week treatment period. Animals receiving DEN and 2-AAF tended to gain less weight (Figure 2A). This difference was significant in the Dmg+13wks-LsPF group compared to the control (Ctl) group in the second half of treatment (*p* < 0.05) and throughout treatment in the Dmg+7wks-LsPF and Dmg+13wks-LsPF groups compared to the Ctl group (*p* < 0.05 and *p* < 0.001, respectively) and Dmg+13wks-LsPF compared to LsPF (*p* < 0.05) (Figure 2B).

In contrast to the Ctl animals, the final weight of all groups was significantly lower in the damage treatment (Dmg) without (*p* < 0.05) or with LsPF (*p* < 0.001) (Figure 2C). No mortality was observed in any of the animals in the experimental groups during the study period.

### 2.4. Liver Function Markers Are Elevated in All Damage Induction Groups

Table 1 shows the average values of the biochemical serum tests in the experimental groups. In the group of animals that received LsPF alone, total bilirubin and total proteins were significantly altered compared to Ctl (*p* < 0.05) but remained within the normal range (0.02–0.42 and 5.76–6.94, respectively). LsPF also increased alanine aminotransferase (ALT) and glucose levels (*p* < 0.05), but slightly above the normal range (22.68–45.64 and 39.55–137.06, respectively). In contrast to the Ctl and LsPF groups, hepatic function markers were elevated in all Dmg-treated groups. ALT, aspartate aminotransferase (AST), alkaline phosphatase (ALKP), total bilirubin, and gamma-glutamyl transferase (GGT) (*p* < 0.05 − 0.001), as well as cholesterol (*p* < 0.05 − 0.001), and high-density lipoprotein (HDL-C) (*p* < 0.001). In addition, the total protein concentration was increased in the Dmg and Dmg+7wks-LsPF groups compared to the untreated animals (*p* < 0.05) but was close to the normal range. Interestingly, serum glucose was elevated in the samples from the LsPF and Dmg+7wks-LsPF animals compared to those from the control group (*p* < 0.05), and the latter was also elevated compared to the Dmg group (*p* < 0.05) [36].

### 2.5. Administration of DEN and 2-AAF Induces Nodule Formation and Hepatomegaly

**Figure 3:**
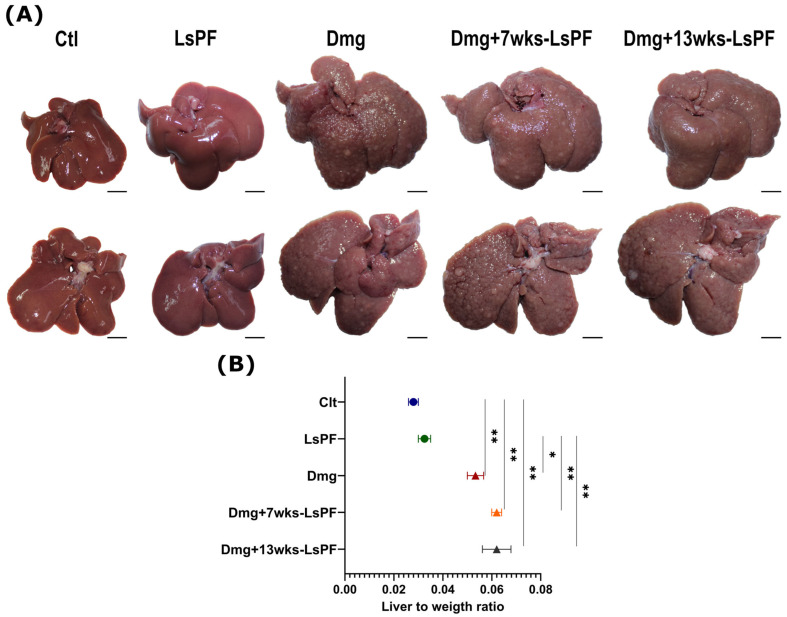
Formation of liver nodules and hepatomegaly due to damage treatment. (**A**) Representative images of the livers of the control and the treated groups [Scale bar: 1 cm]. (**B**) Liver to weight ratio (± SEM) [Calculated as liver weight/animal body weight]. Statistical significance was analyzed using the Mann–Whitney test. * *p* < 0.05; ** *p* < 0.01. [Ctl, Control; LsPF, *Lophocereus schottii* Polar Fraction; Dmg, Damage; wks, weeks].

The livers of the Ctl group showed a bright brown reddish coloration, smooth shiny surface, and even lobular margins. In the groups treated with Dmg, the coloration of the tissue was pale, and the edges of the lobules were uneven. In addition, they were hard to the touch, and well-differentiated nodules were visible on their surface (Figure 3A). In all Dmg-treated groups, the liver-to-weight ratio was significantly increased, and the mean liver-to-weight ratio was more than twice that of the Ctl group (*p* < 0.01) and the LsPF group (*p* < 0.05 − 0.01) (Figure 3B).

### 2.6. Damage Treatment Induces Histological Changes and Fibrosis

**Figure 4:**
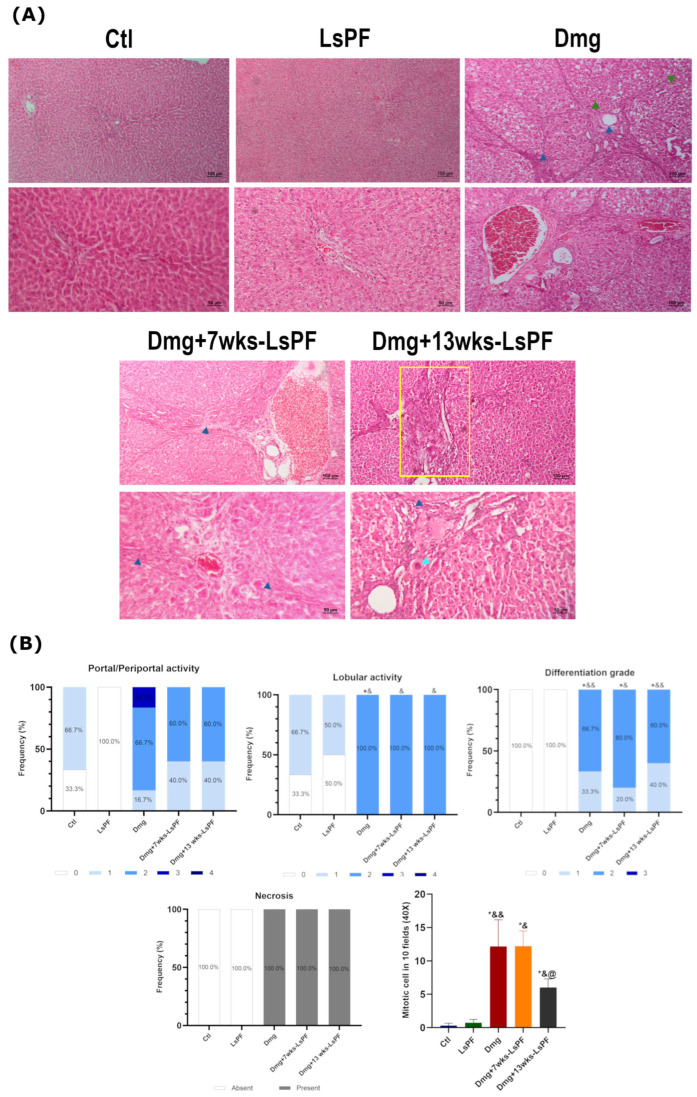
Damage treatment increases the degree of lobular activity and differentiation, necrosis, and mitotic rate in liver tissue. (**A**) Representative images of H&E-stained liver tissue from all (Images: Top row and bottom row 10× and 20× objectives, respectively. (Blue arrowhead: inflammatory infiltrate; clear blue arrowhead: nucleomegaly; green arrowhead: well-differentiated nodule; yellow rectangle: portal inflammation). (**B**) Frequency (expressed as percentage) of portal/periportal and lobular activity levels, differentiation grades, and the presence or absence of necrosis in the animals of the different groups. The mean number of mitotic cells (± SEM) found in 10 aleatory fields at 40×. Statistical significance was analyzed using the Mann–Whitney test. * *p* < 0.05 compared to the Ctl group; ^&^ *p* < 0.05 compared to the LsPF group; ^&&^ *p* < 0.01 compared to the LsPF group. ^@^ *p* < 0.05 compared to the Dmg+7wks-LsPF. [Ctl, Control; LsPF, *Lophocereus schottii* Polar Fraction; Dmg, Damage; wks, weeks].

Hematoxylin & Eosin (H&E) staining of liver sections from the Ctl group showed normal architecture of the hepatic lobules (lobular activity between grades 0 and 1), central veins, portal triads (portal activity between grades 0 and 1), and absence of necrosis. In contrast, the hepatic architecture of all DEN+2-AAF-treated groups showed dysplastic nodules, inflammatory infiltrates, cells with nuclear atypia, significantly increased lobular activity (grade 2, *p* < 0.05), cell differentiation (grades 1 and 2, *p* < 0.05 − 0.01), and mitotic cell counts. Remarkably, administration of the polar fraction for 13 weeks together with Dmg treatment results in a reduction in the mitotic rate in the hepatic cells (*p* < 0.05 − 0.01) (also significantly reduced compared to Dmg+7wks-LsPF, *p* < 0.05) (Figure 4A,B).

**Figure 5:**
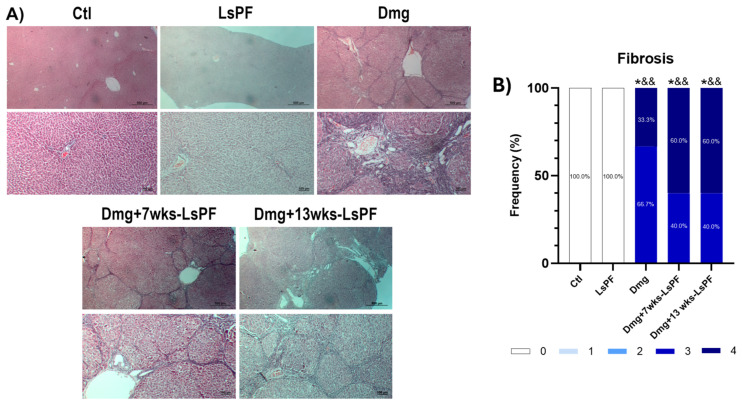
Treatment with DEN and 2-AAF induces advanced fibrosis. (**A**) Representative images of liver tissue stained with Masson (Images: top row and bottom row 10× and 20× objectives, respectively). (**B**) Frequency (expressed as percentage) of fibrosis scores in the animals of the different groups. Statistical significance was analyzed by comparing fibrosis scores using the Mann–Whitney test. * *p* < 0.05 compared to the Ctl group; ^&&^ *p* < 0.01 compared to the LsPF group. [Ctl, Control; LsPF, *Lophocereus schottii* Polar Fraction; Dmg, Damage; wks, weeks].

In addition, Masson’s trichrome staining visualized the clear formation of fibrous septa induced by Dmg treatment in all administered animals (Figure 5A). The degree of fibrosis was similar in the Dmg, Dmg+7wks-LsPF, and Dmg+13wks-LsPF groups (grades 3 and 4) and significantly higher than in the Ctl and LsPF groups (grade 0 in 100% of samples) (*p* < 0.05 − 0.01) (Figure 5B).

### 2.7. LsPF May Have a Better Effect on Gene Expression in Already Damaged Tissue

**Figure 6:**
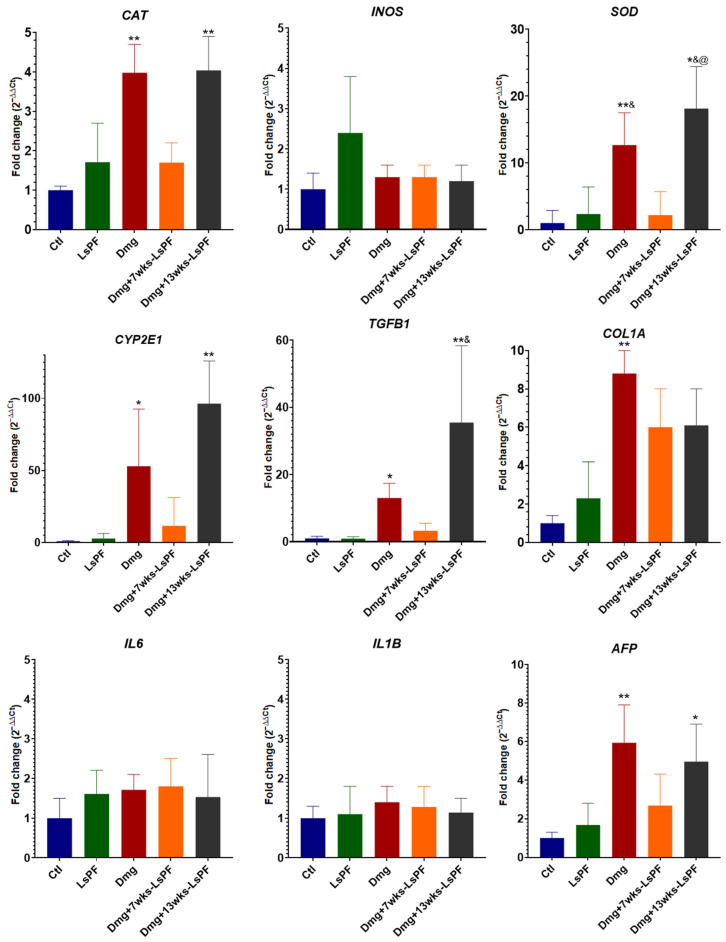
Seven weeks of treatment with LsPF significantly alters gene expression compared to other damage groups. mRNA expression levels in liver tissue, data expressed as fold change (2^−ΔΔCt^) based on ΔCt values compared to CTL group. Data was normalized to the expression of the *RPL41* gene. Statistical significance was analyzed by comparing ΔCt values using the Mann–Whitney test. * *p* < 0.05 compared to the Ctl group; ** *p* < 0.01 compared to the Ctl group; ^&^ *p* < 0.05 compared to the LsPF group; ^@^ *p* < 0.05 compared to the Dmg+7wks-LsPF group. [Ctl, Control; LsPF, *Lophocereus schottii* Polar Fraction; Dmg, Damage; wks, weeks].

Damage treatment significantly increased the expression of *CAT*, *SOD*, *CYP2E1*, *TGFB1*, and *AFP* in the Dmg and Dmg+13wks-LsPF groups. In the Dmg+7wks-LsPF group, the expression of these genes tended to be higher than in the Ctl and LsPF groups, but also lower than in the other damage groups, with no significant differences in either case. *COL1A* mRNA levels were significantly increased only in the Dmg group. No significant differences were found in the expression of *INOS*, *IL6,* and *IL1B* in any of the groups (Figure 6).

## 3. Discussion

Orozco-Barocio et al. (2022) [27] have previously demonstrated that LsPF has a cytotoxic effect, which was stronger in murine L5178Y lymphoma cells compared to healthy splenocytes. However, the effect was found to be particularly effective in cells undergoing mitosis (L5178Y cells and splenocytes stimulated with Concanavalin A) [27]. In the current study, the viability of the cell lines Huh7 and HepG2 was significantly reduced by a dose of 125 µg/mL LsPF. This effect can be attributed to the secondary metabolites contained in LsPF. 97.15% are alkaloids [lophocerine, peyophorine, phenylalanine-betaxanthin and 2,17-didecarboxy-neobetanin], 0.23% are flavonoids [kaempferol xyloside, kaempferol 3-O-glucoside (astragalin), cyanidin 3-O-glucoside (kuromanine), kaempferol 3-O-xylosyl-glucoside], 0.07% terpenoids [phytofluene and alpha-tocotrienol], and 2.48% of phenolic acids [coumaroyl malic acid] or of unknown type [27]. 

Senita cactus contains unusual alkaloids, including pilocereine, piloceredine, and lophocerine [40,41,42], as well as lupeol, lophenol, and schottenol. The presence of unusual sterols in this cactus is due to the accumulation of intermediate forms caused by putative disruptions in sterol biosynthetic pathways [43]. Lophocerine and peyophorine, together with lycorine, berberine, tetandrine, and colchicine; these four compounds reduce HCC cell viability, proliferation, and/or migration [44]. In addition, phenylalanine-betaxanthin (phenylalanine-bx) and 2,17-didecarboxy-neobetanin are betalains, which are a type of compounds that induce chemoprotective activity by increasing apoptosis [45].

Of the flavonoids in LsPF, kaempferol 3-O-glucoside inhibits the proliferation of Huh7 and HepG2 cells by regulating the apoptosis signaling pathway by decreasing Bcl-2 expression, and increasing the expression of Bax, cleaved caspase-3, cleaved caspase-8, and cleaved caspase-9. Furthermore, in vivo and in vitro assays have shown that it reduces HCC cells’ proliferation, inhibits glycolysis, promotes the accumulation of ROS, and causes growth arrest. Furthermore, its administration by gavage reduces the proliferation of Huh7 HCC xenografts in nude mice [46].

Cyanidin-3-O-glucoside is present in the LsPF at a small concentration of 0.02%. However, this compound has been found to slightly reduce the viability of HepG2 cells. The mechanism for this effect remains unclear, particularly since cyanidin-3-O-glucoside is known to increase telomerase reverse transcriptase (*hTERT)* expression by upregulating nuclear factor erythroid 2-related factor 2 (Nrf2) in the same cell line [47,48]. Of note is the study by Matboli et al. (2021) [49], in which the administration of cyanidin-3-O-glucoside reduced the size and number of dysplastic nodules in the precancerous lesion of the liver in a model of DEN+2-AAF (DEN once weekly for three consecutive weeks and a single dose of injected 2-AAF) induced liver damage. The reduction in nodules was achieved by modulating cell cycle progression, resulting in decreased cellular proliferation [49].

The reduction in viability with LsPF was much more pronounced in HepG2 cells than in Huh7 cells. Huh7 cells have higher levels of phase I enzyme activities (CYP1A1/2, CYP2A6, CYP2B6, CYP2C8/9, CYP2E1, and CYP3A4) than other hepatic cell lines, including HepG2 [50]. Alkaloids are able to inhibit the proliferation of Huh7 and HepG2 cells (whose viability tends to be lower compared to Huh7 cells), compared to LX-2 cells [51]. The different susceptibility of HepG2 cells compared to Huh7 cells may be due to the genetic differences between the two cell lines, as has been previously reported [52,53,54].

In the present in vivo model, survival rates reached 100% in all experimental groups during the entire 13-week treatment period. This result contrasts with a previous study that used an identical regimen of DEN and 2-AAF in Wistar rats. That study reported an 18-week survival rate of 62.5% and observed histopathological changes in the lung indicative of metastasis [11]. To rule out possible metastasis and minimize animal suffering, euthanasia was performed at week 13. This endpoint was chosen based on the observation of a progressive decrease in body weight that began in the fourth week and continued thereafter. Furthermore, this time frame is supported by a previous study in Fisher rats _a strain more susceptible to HCC_ in, which complete hepatic tumor invasion was documented after 12 weeks following the same treatment protocol [55].

Induction of liver damage in this study resulted in a significant reduction in body weight, an increase in liver weight and serum biochemical markers. Administration of LsPF did not attenuate these changes. Previous reports confirm that DEN either alone or in synergy with 2-AAF, promotes weight loss and hepatomegaly, an effect likely mediated by liver injury-induced disturbances in energy metabolism [56,57,58,59,60,61]. Although not statistically significant, the liver-to-body weight ratio was increased in the damaged groups treated concurrently with LsPF for 7 and 13 weeks compared to the damage-only group. This observed hepatomegaly could be due to more severe fibrosis scores in the groups with damage plus LsPF treatment compared to the damage-only group (grade 4: 60% vs. 33.3%, respectively), alongside a trend towards higher biochemical marker values. Consequently, further studies need to be conducted to investigate a synergistic hepatotoxic effect between the administration of LsPF with DEN and 2-AAF.

Nevertheless, the histologic and gene expression analyses showed no significant differences between the LsPF-treated rats and the control group, suggesting that the extract does not exhibit hepatotoxicity. However, biochemical analysis showed slight elevations of ALT and glucose in serum, which could be a consequence of the long-term treatment. Previous reports have shown that the administration of plant extracts increased ALT [37,62,63,64]. The major metabolites in the fraction are alkaloids known for their hypoglycemic activity [65,66]; however, there is a report that cathinone, an alkaloid of *Catha edulis* (Khat), increases glucose levels, probably through its sympathomimetic glycogenolytic activity [67,68]. Probably some unidentified alkaloids (which represent 2.4% of the actives in the LsPF) in the fraction may have a similar effect. LsPF itself increased ALT (52.50 ± 8.50) and glucose (146.0 ± 32.32), which were slightly above their normal range values (22.68–45.64 and 39.55–137.06). This may indicate that long-term administration of the fraction is not optimal or that lower doses need to be tested for therapeutic use. However, it is important to emphasize that histological analysis revealed no liver damage in the tissue.

Although a previous study by Orozco-Barocio has demonstrated that intratumoral and intramuscular administration of the ethanolic extract of *L. schottii* reduced tumor growth in a solid L5178Y murine lymphoma model [26], the administration of LsPF in this model of hepatic damage did not generally ameliorate the macroscopic and histologic changes induced by the treatment. It is important to emphasize that the intragastric route was used in this study, because it allows for safe testing of new compounds [69]. However, a disadvantage is that the bioavailability of the compounds contained in the extract may be affected by the amount of drug absorbed through the intestinal epithelium, and their concentration may be significantly reduced before they reach the systemic circulation [70]. Intravenous administration may have allowed a higher dose, and intramuscular administration a better absorption [71]. For these reasons, in future models with LsFP, it may be better to evaluate the outcome with the later routes of administration. Another consideration is that the studied ethanolic extract was in a single solid tumor model that allows direct delivery to the desired site, whereas the DEN+2-AAF-induced model can generate tumors at multiple tissue sites. In addition, histological analysis showed that the administration of LsPF concurrent with the damage-inducing treatment over the 13-week period tended to reduce the number of mitotic cells compared to the other damage groups. This phenomenon suggests that seven weeks post-damage treatment, the tissue alteration may have progressed to a stage where LsPF can no longer exert this effect. This observed reduction in mitotic cells is consistent with the aforementioned cytotoxic effect of LsPF on mitotic cells.

The expression of *AFP*, *TGFB1*, *CYP2E1*, *CAT*, and *SOD* was significantly increased in the livers of the Dmg and Dmg+13wks-LsPF groups. In the fetal liver, *AFP* is highly expressed and dramatically suppressed soon after birth [72]. Abnormal expression of alpha-fetoprotein (AFP) is mainly found in liver cancer. The re-activation of its expression in liver cancer is the result of an alteration of the systematic transcriptome, and its high expression is associated with excessive activation of genes that control cell growth or the cell cycle [73]. While TGF-β plays a complex role in cancer, its elevated levels are thought to promote tumor development through effects on tumor stromal cells and local immune cells, or possibly on tumor cells that have evolved mechanisms to evade the cell-autonomous tumor suppressor activities of TGF-β. In certain cancer cell types, inactivation of p53 may contribute to the deficit in TGF-β antiproliferative activity. A model using *Trp53^KO^* mice showed that TGF-β and p53 cooperate in the regulation of certain target genes, including *AFP*. p53 appears to be required for TGF-β/Smad-mediated transcriptional repression of *AFP* [74,75,76]. This connection may explain how dysregulation of TGF-β affects AFP in our model. However, in a previous work by our group, using a similar model but with damage induction lasting 18 weeks, there were no changes in the expression of AFP [11]. Further studies are being conducted to clarify whether the timing of chemical damage induction could be the cause of these differences.

In addition, damage induction increased the expression of cytochrome P450 family 2 subfamily E member 1 (CYP2E1). It has been previously reported that the interaction of TGF-β1 and CYP2E1 increases hepatocyte toxicity in vitro, probably through an increase in oxidative stress, leading to mitochondrial membrane damage and loss of membrane potential, followed by apoptosis and necrosis [77]. In our damage model, this effect may have been reflected in the histologic findings of necrotic sites in the tissue, which were present in all groups with liver damage.

CYP2E1 is found mainly in the liver and is expressed primarily in hepatocytes; however, significant amounts are also found in Kupffer cells; it is inducible in both cell types. This enzyme metabolizes a variety of small, hydrophobic substrates and drugs, including procarcinogens such as nitrosamines and azo compounds, which are effective substrates for this enzyme. The toxicity of these substrates is enhanced by the induction of CYP2E1 [78]. The bioactivation of DEN is influenced by several P450 isozymes, including CYP2E1 [79,80,81]. Apart from the fact that CYP2E1 is involved in DEN-induced hepatocarcinogenesis in vivo [82], higher constitutive CYP2E1 activity contributes more to DEN-induced hepatocarcinogenesis [83]. In this case, the increase in *CYP2E1* could play an important role in the development of damage in the model used in the present study; the expression of this gene tended to be lower in Dmg+7wks-LsPF compared to Dmg and Dmg+13wks-LsPF.

CYP2E1 activity is significantly elevated in human fibrotic tissue. This correlation is supported by animal models. In rats treated with DEN, higher innate CYP2E1 activity was associated with more severe liver fibrosis. Furthermore, inhibition of CYP2E1 during DEN treatment attenuated the development of hepatic fibrosis, and an inverse correlation was observed between the degree of enzymatic inhibition and the extent of fibrotic damage. Therefore, high CYP2E1 activity constitutes a risk factor for nitrosamine-induced hepatofibrogenesis [84]. This mechanism may help to link the increase in *CYP2E1* expression to the development of high-grade fibrosis (3 to 4) in our model.

The catalytic cycle of CYP2E1 tends to uncouple, leading to the production of ROS. Nrf2, a redox-sensitive transcription factor, increases the expression of genes encoding cytoprotective products, such as antioxidants and phase II conjugation enzymes; at the same time, dying cells release pro-inflammatory signals. Together, these signals promote the regenerative proliferation of hepatocytes. However, chronic activation of these molecular signals can dysregulate cellular proliferation, whereby the cytoprotective cellular mechanisms can lead to premalignant and malignant lesions [85]. There is limited data on the effects of cacti or their fruit extracts on these molecules. Only a few reports exist, such as one indicating that the peel extract of red pitaya (*Hylocereus polyrhizus* (Weber) Britt. & Rose) inhibits CYP2E1 and Nrf2 protein expression [86].

The isoquinoline alkaloids of plants interact with drug-metabolizing cytochrome P450 enzymes and in some cases can inhibit their activity [87,88]. There are few data on the metabolism of cactus alkaloids by these enzymes, and most of this data comes from studies with drosophilids. Cytochrome P450 enzymes are involved in the detoxification and tolerance of cactus alkaloids in drosophilids; exposure to alkaloid-containing cacti increases the total amount of cytochrome P450 in desert Drosophila. Interestingly, the species with the highest cytochrome P450 content showed the lowest basal activity [89,90,91]. Moreover, *D. pachea* is the only species that can tolerate a high concentration of isoquinoline alkaloids (lophocereine and pilocereine) found in its tissues [92].

Further studies are necessary to determine the effect of LsPF on the activity of CYPs in human cells. This investigation is particularly warranted given that the alkaloids in *L. schottii* possess a unique compositional profile that distinguishes them from other alkaloid classes. Based on the observations in our model and reports in drosophilids, it is possible that *CYP2E1* expression increased by the seventh week of damage. The initiation of LsFP administration at this time point may then have favored the observed tendency toward reduced enzyme expression compared to other damage groups. However, more studies that include an analysis of different CYP 450 isoforms need to be performed.

In contrast to healthy cells, cancer cells often have high levels of ROS and altered concentrations of antioxidant molecules. Of the latter, SODs play a crucial role in the cellular defense against oxidative stress by converting the O_2_^−^ into H_2_O_2_. There are three main forms of SOD, including the copper-zinc superoxide dismutase (Cu/Zn-SOD or SOD1) [93]. Catalases, on the other hand, exert their catalytic activity mainly by splitting H_2_O_2_ into water and molecular oxygen. Frequently, the expression of catalase is decreased in human tumor tissue, but increased expression of catalase has also been observed in tumors of gastric carcinoma, skin cancer, and chronic myeloid leukemia [94]. In liver cancer, however, there are mainly reports of decreased expression of CAT and SOD [93,95,96].

Fahim et al. (2023) show how DEN administration to Wistar rats increases the activity of CYP2E1 and decreases the activity of CAT and SOD in liver tissue [97]. According to our observation, overexpression of *CYP2E1* could lead to increased activity in tissues as found by Fahim et al. However, the overexpression of *CAT* and *SOD* in our model is not consistent with their results, as they also contrast with another study in which the induction of HCC by DEN decreased the expression of *CAT* and *SOD1* [57]. There are in vitro reports of increased CAT and SOD expression in HCC cell lines, such as overexpression of *CYP2E1*, which increased *CAT* expression in HepG2 cells [98]; and *SOD1* expression in a CYP2E1-expressing HepG2 transgenic cell line [99]. The increased expression of *CAT* and *SOD* may be a mechanism that confers resistance to prooxidants in cells as an adaptive response to CYP2E1-mediated oxidative stress. The difference between our results and those of other studies could be due to the different methods of HCC induction, as in some of them only DEN is used, while in our study we use 2-AAF, about whose effect on gene expression there is few data; and even less about the effect on different periods of damage induction.

In a previous study, differences in gene expression were found when Wistar rats were treated with DEN and 2-AFF for 18 weeks. At this time of treatment, gene expression was observed in the damage group compared to the control group [11]. In this study, after 13 weeks of treatment with DEN and 2-AAF, opposite results were observed with respect to the expression of these genes, with an increase in *CYP2E, CAT, SOD*, and *AFP*, and a decrease in *IL-6*. It is possible that prolonged treatment leads to epigenetic mutations reflected in a decreased expression of *CYP2E1*, *CAT*, *SOD*, and a normalization of *AFP* expression. It is likely that after 13 weeks there is still a higher metabolic activity of CYP2E1, with an increase in CAT and SOD attempting to compensate for the damage induced by DEN and 2-AAF treatment. Further studies are needed to determine the effects of treatment duration on the observed changes in gene expression.

Regarding the effects of LsPF on the expression of genes involved in carcinogenesis, the levels of expression of *CAT*, *SOD*, *CYP2E1*, *TGFB1*, and *AFP* by the damaged treatment alone or together with LsPF were similar during the 13 weeks. However, when the fraction was administered from the seventh week after the onset of damage induction, it tended to be reduced compared to the other damaged groups; nevertheless, the expression levels in the Dmg+7wks-LsPF were not significantly different from those of the other four groups.

In conclusion, LsPF did not significantly improve macroscopic, biochemical or histologic outcomes, neither when co-administered concomitantly or after 7 weeks of treatment with DEN and 2-AAF treatment. However, co-administration of LsPF with the damage treatment decreased the number of mitotic hepatocytes. Moreover, when administered after 7 weeks of HCC induction, LsPF modulated the expression of genes related to liver carcinogenesis, including *SOD*, *CAT*, *CYP2E1*, *TGFB1*, *AFP*, and *COL1A.* These results suggest that LsPF may exert a modulatory effect on the expression of genes involved in HCC progression. In our model, the timing of treatment initiation appeared to be the factor influencing this trend of gene expression and the reduction in hepatocyte mitosis. LsPF might have more favorable therapeutic outcomes in a model with less severe injury or when initiated after cancer induction. In addition, it may be ideal to change the route of administration from intragastric to intraperitoneal administration for future studies and in larger experimental groups.

### Limitations of the Study

The experiments in this study used a rat model with chemically induced HCC, a method that may not fully reflect the complex tumor microenvironment of human HCC, which is primarily etiologically associated with chronic viral hepatitis B and C infection. In addition, the lack of significant improvements in macroscopic, biochemical, and histologic endpoints limits the robustness of the possibly therapeutic applications. Although a significant reduction in mitotic cells was observed in the group treated with LsPF for a prolonged period (13 weeks) compared to the other damage groups, this finding was not accompanied by a reduction in tumor burden. Furthermore, this study did not use a longitudinal study design to characterize the temporal evolution of molecular changes in this animal model. Such an analysis, using a comprehensive panel of biomarkers covering the major mechanisms of hepatocarcinogenesis, including pathways of immune response, would be highly informative. Undoubtedly, such an investigation could elucidate the molecular basis of the observed expression changes in genes such as *SOD*, *CAT*, *CYP2E1*, *TGFB1*, *AFP*, and *COL1A* in all damage groups.

Nevertheless, it is important to report these results as this catus is used in traditional medicine for the treatment of cancer. Although the conclusions of the present study were not definitive, it is plausible that alternative dosing regimens or routes of administration may offer therapeutic benefits. Therefore, further studies are required to evaluate these parameters.

## 4. Materials and Methods

### 4.1. Preparation of the Polar Fraction of L. schottii

The polar fraction (LsPF) was prepared as previously described by Orozco-Barocio et al. (2022) [27]. In brief, the ethanolic extract was obtained by extracting small dry pieces of *L. schottii* stem in absolute ethanol (1:10 *w*/*v*) with stirring for 48 h at room temperature. The polar extract was obtained by fractionation with hexane at a ratio of 1:2 (ethanolic extract–hexane), by separating it from a non-polar fraction using a separatory funnel. The LsPF obtained from the ethanolic extract was concentrated under low pressure in a rotary evaporator (Mod. RE47, Yamato Scientific Co., Ltd, Tokyo, Japan) and stored in dark refrigerated bottles [27]. The obtained yield (*w*/*v*) of polar fraction was around 0.65%.

### 4.2. Identification of Compounds from the Polar Fraction Extract by UPLC-MS

UPLC-MS analysis was performed in accordance with the previously published method [27]. This analysis was applied to four separate batches of LsPF; each obtained during a distinct season. In brief, 1 mg of the sample was dissolved in 1 mL of HPLC methanol as solvent and filtered through a 0.45 μm syringe filter for analysis. UPLC-MS analysis was performed using an ACQUITY UPLC system liquid chromatography instrument coupled to a Waters (Milford, MA, USA) QDA^®^ mass detector ACQUITY UPLC CORTECS^®^ C18 column 1.6 μm 3.0 × 100 mm). The specific conditions are described elsewhere [27].

Data analysis was performed using MassHunter Qualitative Analysis B.06.00 (Agilent Technologies, Santa Clara, CA, USA). Compounds characterization was performed by generating candidate formulas with a mass accuracy limit of 10 ppm. The characterization was performed with four different batches of LsFP. The complete list of identified secondary metabolites from the polar extract is shown in Supplementary Table S1 and described in Orozco-Barocio et al. (2022) [27].

### 4.3. Qualitative Determination of Phytochemicals in the Polar Fraction of L. schottii

A qualitative determination of the presence of alkaloids, steroids, flavonoids, terpenoids, phenols, and quinones is also performed using the methods described by Jasnie (2009) [100] and Jara-Beltrán (2013) [101]. The qualitative analysis was performed after each preparation of a new LsPF extract. The methods used are listed in the Supplementary Table S2: Precipitation of alkaloids with Mayer’s reagent; Shinoda test for flavones; test for terpenoids with sulfuric acid; ferric chloride test for phenols; potassium hydroxide test for quinones; and analysis of steroids and triterpenoids with thin-layer chromatography (mobile phase: petroleum ether/ethyl ether/acetic acid 80:20:1). This analysis was performed after each LsPF extraction.

### 4.4. Effect of the Polar Fraction of L. schottii on the Viability of Liver Cancer Cell Lines

Huh7 (kindly provided by Dr. J. Liang) or HepG2 cells were maintained at 37 °C and 5% CO_2_ in Dulbecco’s Modified Eagle Medium (Gibco, Life Technologies Corporation, Grand Island, NY, USA) supplemented with 10% fetal bovine serum (ScienCell, Research laboratories, Carlsbad, CA, USA) and 1% antibiotic–antimycotic (Gibco, Life Technologies Corporation, Grand Island, NY, USA). Fresh medium was added every third day. For viability assays, cells were seeded at a density of 0.01 × 10^6^ cells per well in 96-well plates. Cells were treated with 0 µg/mL, 25 µg/mL, 50 µg/mL, 75 µg/mL, 100 µg/mL, and 125 µg/mL LsPF during 72 h. PrestoBlue^TM^ Cell Viability Reagent (Invitrogen, Thermo Fisher Scientific, Eugene, OR, USA) was used to measure viability after treatment according to the manufacturer instructions.

### 4.5. In Vivo Chemical Induction of Liver Cancer and Treatment with the Polar Fraction of L. schottii

The protocol was approved by the Ethics, Research and Biosafety Committee of the Centro Universitario de Ciencias de la Salud (CUCS), Universidad de Guadalajara (UdG) (approval number: CI-05023). Healthy male Wistar rats (190–235 g) with no previous health problems and/or treatments were obtained from the Centro de Investigación Biomédica de Occidente, Instituto Mexicano del Seguro Social. The animals were housed and cared for according to the official Mexican standard (NOM-062-ZOO-1999), which establishes the technical specifications for the production, care, and use of laboratory animals [102] and the code of conduct for the housing and care of animals bred, supplied or used for scientific purposes [103].

Before and during the experimental protocol, the animals were kept under controlled environmental conditions (room temperature between 22 °C and 25 °C, 12-hour light-dark cycle, with free access to standard food and water). The cages were designed to avoid contact with other animals or prey. The cages had transparent solid walls (20 cm high) and floors (387 cm^2^ per animal of 400–500 g) and a removable mesh cover. The cages were exposed to constant ventilation and lighting and were located in the same room so that all animals were in the same conditions. The food and water came from the same sources. No additional environmental enrichment was provided.

Liver damage was induced by chronic administration of DEN (Sigma-Aldrich, Inc, St. Louis, MO, USA, 68178) and 2-AAF (Sigma-Aldrich, Inc, St. Louis, MO, USA, 68178). Animals receiving noxious treatment were specifically monitored for adverse effects such as weight loss, skin disease and respiratory problems. Humane endpoints were monitored throughout the protocol, such as signs of chronic pain (using the Rat Grimace Scale [104]), severe emaciation, respiratory problems, signs of disease, etc. If any of these signs occurred and the animals were irreversibly affected, they were sacrificed

**Figure 7:**
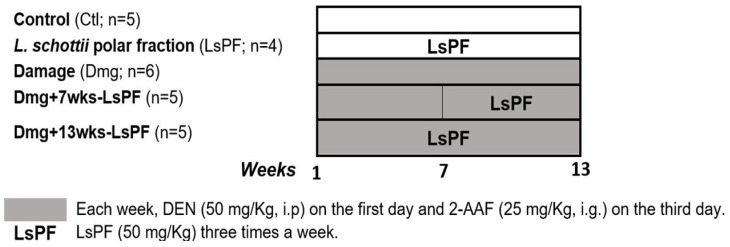
Treatment regimen for induction of liver damage in Wistar rats and treatment with LsPF. [Ctl, Control; LsPF, *Lophocereus schottii* Polar Fraction; Dmg, Damage; wks, weeks; DEN, diethylnitrosamine; 2-AAF, N-2-fluorenylacetamide; i.p. intraperitoneal; i.g, intragastric; mg, milligrams; Kg, kilograms].

25 male Wistar rats (180–200 g body weight) were randomly divided into five groups: (1) Control group (Ctl); (2) Damage group (Dmg); (3) Ls PF group (LsPF); (4) Damage with LsPF for seven weeks (Dmg+7wks-LsPF); and (5) Damage with LsPF for 13 weeks (Dmg+13wks-LsPF). To induce injury, animals were treated weekly intraperitoneally with DEN (50 mg/Kg, i.p.) on the first day and intragastrical with 2-AAF (25 mg/Kg, i.g.) on the third day every week for 13 weeks. The Dmg+7wks-LsPF and Dmg+13wks-LsPF groups received LsPF (50 mg/Kg, i.g.) three times a week from the 7th to the 13th week and from the 1st to the 13th week, respectively (Figure 7).

At the end of treatment, the animals were anesthetized for blood sampling by cardiac puncture and the livers were removed and washed in saline solution. Portions of the liver tissue were fixed in 4% formaldehyde for histological analysis or stored at −80 °C for RNA extraction.

### 4.6. Serum Biochemistry

The serum was obtained by centrifugation of the blood at 3500 rpm for 10 minutes to determine glucose, urea, creatinine, total cholesterol, triglycerides, HDL-C, total protein, AST, ALKP, GGT, and ALT (Orto Clinical Diagnostics) by dry chemistry with the VITROS 350^®^ Analyzer (Ortho-Clinical Diagnostics, Rochester, NY, USA).

### 4.7. Histological Analysis

According to the supplier’s instructions, formaldehyde-fixed tissues were embedded in paraffin blocks and 5 µm paraffin sections were cut and stained with H&E and Masson trichrome staining. Images were captured and analyzed using a bright-field microscope (Carl Zeiss, Primo Star, Göttingen, Germany).

Histopathologic analysis was performed by two independent pathologists using the variables listed in Table 2. The degree of activity and fibrosis stage were assessed using the Scheuer system for grading and classifying chronic hepatitis [105].

### 4.8. Analysis of Gene Expression

Total RNA was extracted with 500 µL of Trizol (Invitrogen, Carlsbad, CA, USA) per 100 mg of tissue. The extraction protocol was performed according to the manufacturer’s instructions. RNA integrity and concentration were determined using a NanoDrop^TM^ One/One^C^ spectrophotometer (Thermo Scientific, Waltham, MA, USA) at wavelengths of 260/280/230. The RNA was stored at −80 °C until use.

Reverse transcription of RNA to cDNA was performed using an M-MLV RT enzyme (Invitrogen, 200 U/μL) according to the manufacturer’s instructions. Subsequently, real-time quantitative PCR (qPCR) was performed using the LightCycler^®^96 instrument (Roche, Mannheim, Germany) and FastStart Universal SYBR Green Master (Roche; Mannheim, Germany) according to the manufacturer’s instructions. For gene expression analysis, the primers and qPCR conditions indicated by Sánchez-Meza et al. (2023) [11] were followed, using *RPL41* as the constitutive gene [11] and the primer sequences are listed in Table 3. Data was extracted using LightCycler^®^ 96 software (version 1.1.0.1320). Gene expression changes are represented as fold changes and were calculated using the 2^−ΔΔCt^ method.

## Figures and Tables

**Table 1:** Biochemical serum values of experimental groups.

	Reference Values	Ctl	LsPF	Dmg	Dmg+7wks-LsPF	Dmg+13wks-LsPF
Biochemical markers						
ALT (U/L)	22.68–45.64 ^a^	39.80 ± 1.715	52.50 ± 4.252 *	162. 8 ± 24.42 **^&^	216.2 ± 25.90 **^&^	274.4 ± 42.31 **^&$^
AST (U/L)	85.71–213.33 ^a^	173.0 ± 8.88	170.3 ± 28.40	357.2 ± 31.83 **^&&^	376.8 ± 33.49 **^&^	485.8± 70.96 **^&^
ALKP (U/L)	81.16–209.65 ^a^	151.8 ± 7.372	197.8 ± 32.67	563.7 ± 46.71 **^&&^	619.0 ± 61.98 **^&^	423.00 ± 39.35 **^&^
Albumin (g/dL)	3.4–4.8 ^a^	3.700 ± 0.0707	4.025 ± 0.1493	3.783 ± 0.1579	3.66 ± 0.0748	3.560 ± 0.2542
Total bilirubin (mg/dL)	0.02–0.42 ^a^	0.2400 ± 0.02449	0.1250 ± 0.02500 *	1.500 ± 0.7559 **^&&^	0.9400 ± 0.1364 **^&^	2.520 ± 1.027 **^&^
GGT (U/L)	0–6 ^b^	8.000 ± 0.000	8.000 ± 0.000	47.50 ± 8.036 **^&&^	64.40 ± 2.821 **^&&^	80.80 ± 15.70 **^&&^
Total proteins (g/dL)	5.76–6.94 ^a^	6.220 ± 0.0969	6.750 ± 0.2062 *	6.983 ± 0.1470 *	6.680 ± 0.1428 *	6.560 ± 0.2293
Creatinine (mg/dL)	0.34–0.9 ^a^	0.52 ± 0.037	0.63 ± 0.085	0.58 ± 0.031	0.50 ± 0.000	0.48 ± 0.037
Urea (mg/dL)	21.74–48.2 ^a^	48.00 ± 3.597	46.53 ± 2.904	49.17 ± 2.287	47.84 ± 1.326	45.38 ± 2.451
Glucose (mg/dL)	39.55–137.06 ^a^	91.0 ± 9.450	146.0 ± 16.16 *	104.3 ± 7.606^&^	133.0 ± 8.068 *^$^	113.2 ± 9.723
Lipids profile						
Cholesterol (mg/dL)	20–92 ^b^	51.80 ± 1.655	62.00 ± 9.174	111.70 ± 10.49 **^&^	109.00 ± 8.081 **^&^	117.40 ± 10.48 **^&^
Triglycerides (mg/dL)	27–108 ^b^	76.80 ± 8.817	72.00 ± 15.71	96.00 ± 4.619	71.20 ± 8.411	97.20 ± 12.54
HDL-C (mg/dL)	36.26–54.55 ^c^	37.60 ± 1.503	42.00 ± 6.338	68.00 ± 3.812 **^&&^	71.00 ± 3.924 **^&&^	67.60 ± 7.718 **

Values per group expressed as Mean ± SEM. Statistical significance was determined using the Mann–Whitney test. * *p* < 0.05; ** *p* < 0.01 compared to the Ctl group. ^&^ *p* < 0.05; ^&&^ *p* < 0.01 compared to the LsPF group. ^$^ *p* < 0.05 compared to the Dmg group. Reference values for male Wistar rats: ^a^ taken from Patel et al., 2021 [36]; ^b^ taken from Ballesteros-Ramírez et al., 2021 [37]; ^c^ taken from Ihedioha et al., 2013 & Vigneshwar et al., 2021 [38,39]. [Ctl, Control; LsPF, *Lophocereus schottii* Polar Fraction; Dmg, Damage; wks, weeks; U, units; L, liters; g, grams; dL, deciliters; mg, milligrams].

**Table 2:** Variables for the grading and classification of liver damage.

Activity Grade		
Portal/periportal	Lobular	
None	None	0
Portal inflammation alone	Inflammation but no necrosis	1
Mild piecemeal necrosis	Focal necrosis or acidophilic bodies	2
Moderate piecemeal necrosis	Severe focal cell damage	3
Severe piecemeal necrosis	Damage includes bridging necrosis	4
Fibrosis score		
No fibrosis		0
Enlarged, fibrotic portal tracts		1
Periportal fibrosis or portal-portal septa, but intact architecture		2
Fibrosis with architectural distortion, but no obvious cirrhosis		3
Probable or definite cirrhosis		4
Tumoral features		
Differentiation grade		
Well-differentiated *	1	
Moderate differentiation **	2	
Poorly differentiated ***	3	
Other features		
Necrosis	Present/Absent	
Mitosis	Average mitotic cells (ten fields at 40×)	

* Tumor cells resembling mature hepatocytes with minimal to mild nuclear atypia. ** Tumor cells resembling mature hepatocytes with nucleomegaly, nuclear hyperchromasia, and irregular cytoplasmic margins. *** Malignant-appearing tumor cells with marked atypia and pleomorphism.

**Table 3:** Primer sequences used for rt-qPCR.

Primer ID	Sequence
AFP-F	5′-CTTGGTGAAGCAAAAGCCTGAA
AFP-R	5′-GGACCCTCTTCTGTGAAACAGACT
CAT-F	5′-GGAGGCGGGAACCCAATAG
CAT-R	5′-GTGTGCCATCTCGTCAGTGAA
COL1A-F	5′-CAAGATGGTGGCCGTTACTAC
COL1A-R	5′- AGTACTCTCCGCTCTTCCAG
CYP2E1-F	5′- CTTTCCCTCTTCCCATCCTT
CYP2E1 -R	5′- CCCGTCCAGAAAACTCATTC
IL-1B-F	5′- CCAAGCACCTTCTTTTCCTTC
IL-1B-R	5′- GTCAGACAGCACGAGGCATT
IL-6-F	5′- CCACCCACAACAGACCAGTA
IL-6-R	5′- CTCCAGAAGACCAGAGCAGAT
INOS-F	5′- GCCCCTTCAATGGTTGGTAC
INOS-R	5′- AGGCCAGTGTGTGGGTCTC
SOD-F	5′ AATGTGTCCATTGAAGATCGTGTGA
SOD-R	5′ GCTTCCAGCATTTCCAGTCTTTGTA
TGFB-F	5′ TGCTAATGGTGGACCGCAA
TGFB-R	5′ CACTGCTTCCCGAATGTCTGA
RPL41-F	5′ GGCAGAGGTCCAAGTAAACCA
RPL41-R	5′ ATCTCGGCGAGGTGACATTC

## Data Availability

The original contributions presented in this study are included in the article/Supplementary Materials. Further inquiries can be directed to the corresponding author.

## Supplementary Materials

The following supporting information can be downloaded at: https://www.mdpi.com/article/10.3390/ijms26199788/s1.

## Abbreviation

The following abbreviations are used in this manuscript:

HCC	Hepatocellular carcinoma
LsPF	*Lophocereus schottii* Polar Fraction
DEN	Diethylnitrosamine
2-AAF	2-acetylaminofluorene; N-2-fluorenylacetamide
RNA	Ribonucleic acid
SEM	Standard error mean
Ctl	Control
wks	Weeks
Dmg	Damage
ALT	Alanine aminotransferase
AST	Aspartate aminotransferase
ALKP	Alkaline phosphatase
GGT	Gamma-glutamyl transferase
HDL-C	High-density lipoprotein
H&E	Hematoxylin & Eosin
CAT	Catalase
SOD	Superoxide dismutase
CYP2E	Cytochrome P450 family 2 subfamily E member 1
TGFB1	Transforming growth factor beta 1
AFP	Alpha-fetoprotein
mRNA	Messenger RNA
INOS	Inducible nitric oxide synthase
IL6	Interleukin 6
IL1B	Interleukin 1 beta
ROS	Reactive oxygen species
HK2	Hexokinase 2
Nrf2	Nuclear factor erythroid 2-related factor 2
hTERT,	Human telomerase reverse transcriptase
MALAT1	Metastasis-associated lung adenocarcinoma transcript 1
cDNA	Complementary deoxyribonucleic acid
M-MLV RT	Moloney Murine Leukemia Virus reverse transcriptase
