# Supplementary material for: Effects of the Polar Fraction of Lophocereus schottii on Gene Expression and Hepatocyte Proliferation in a Wistar Rat Model of Hepatocellular Carcinoma
Source: Int J Mol Sci. 2025 Oct 8;26(19):9788. doi: 10.3390/ijms26199788 (PMC12524451; doi:10.3390/ijms26199788)
Supplement: Supplementary file 1 [file ijms-26-09788-s001.zip › ijms-3860498-supplementary.pdf]

Effects of the Polar Fraction of *Lophocereus schottii* on Gene Expression and Hepatocyte Proliferation in a Wistar Rat Model of Hepatocellular Carcinoma

Marina Campos-Valdez[1], Jaime Sánchez-Meza[1], Arturo Orozco-Barocio[2], José A. Domínguez-Rosales[1], Juliana Marisol Godínez-Rubí[3], Sarai C. Rodríguez-Reyes[4], Erika Matínez-López[4], Miriam R. Bueno-Topete[1], Manuel A. Castro-García[1], Guillermo M. Zúñiga-González[5], Daniel Ortuño-Sahagún[6] and Laura V. Sánchez-Orozco[1,*]

Table S1. Secondary metabolites present in the polar fraction of the extract of *Lophocereus schottii.*

Peak	RT (min)	% Area	Exp m/z $[M+H]^+$	Molecular Formula	Tentative Compound Identification	Metabolite Class
1	1.2	85.636	250.17	$C_{15}H_{23}NO_2$	Lophocerine	Alkaloid
2	1.21	10.68	250.21	$C_{14}H_{19}NO_3$	Peyophorine	Alkaloid
3	2.24	0.0262	418.25	$C_{20}H_{19}O_{10}$	Kaempferol xiloside	Flavonoid
4	2.46	0.3397	335.28	-	Unknown	-
5	2.53	1.1661	440.8	-	Unknown	-
7	3.61	0.0542	543.92	$C_{40}H_{62}$	Phytofluene	Terpenoid
8	5.33	0.1421	449.19	$C_{21}H_{20}O_{11}$	Kaempferol 3-O-glucoside	Flavonoid
9	6.33	0.0163	425.34	$C_{29}H_{44}O_2$	Alpha-Tocotrienol	Terpenoid
10	7.33	0.0468	108.3	-	Unknown	-
11	9.04	0.1072	227.2	-	-	-
12	9.6	0.0905	279.58	-	Unknown	-
13	10.16	0.0217	450.41	$C_{21}H_{21}O_{11}$	Cyanidin 3-O-glucoside	Flavonoid
14	10.29	0.0349	581.15	$C_{26}H_{28}O_{15}$	Kaempferol 3-O-xylosyl-glucoside	Flavonoid
15	12.33	0.7682	359.33	$C_{18}H_{18}N_2O_6$	Phenylalanine-betaxanthin	Alkaloid
16	12.65	0.0765	281.78	$C_{18}H_{34}O_2$	Coumaroyl malic acid	Phenolic acids
17	12.83	0.0615	461.4	$C_{22}H_{26}N_2O_9$	2,17-didecarboxy-neobetanin	Alkaloid
18	13.29	0.2238	502.19	-	Unknown	-
19	13.68	0.0589	476.21	-	Unknown	-
20	2.6	0.4045	570.24	-	Unknown	-
21	7	0.0433	573.03	-	Unknown	-

Analysis of the UPLC-MS data revealed a consistent quantitative profile of secondary metabolites among the extracts from the four different seasons.

Published in**:**

Orozco-Barocio A, Robles-Rodríguez BS, Camacho-Corona MDR, Méndez-López LF, Godínez-Rubí M, Peregrina-Sandoval J, Rivera G, Rojas Mayorquín AE, Ortuno-Sahagun D. In vitro Anticancer Activity of the Polar Fraction From the Lophocereus schottii Ethanolic Extract. Front Pharmacol. 2022, 13:820381. doi: 10.3389/fphar.2022.820381

Table S2.

Phytochemical compounds	Method	Evidence of presence
Alkaloids	Mayer's reagent	White precipitate
Steroids and triterpenes	Thin-layer chromatography (TLC). Mobile phase: petroleum ether/ethyl ether/acetic acid (80:20:1)	Stains reveled with Liebermann-Buchard reagent
Flavonoids	Shinoda test	Color change to pink, red, violet or orange
Terpenoids	Concentrated sulfuric acid	Color change to black
Phenols	Ferric chloride 5 %	Color change to black or blue
Quinones	Potassium hydroxide 5 %	Color change to purple and red
